# Sex and age affect acute and persisting COVID-19 illness

**DOI:** 10.1038/s41598-023-33150-x

**Published:** 2023-04-13

**Authors:** Anna Vasilevskaya, Asma Mushtaque, Michelle Y. Tsang, Batoul Alwazan, Margaret Herridge, Angela M. Cheung, Maria Carmela Tartaglia

**Affiliations:** 1 Tanz Centre for Research in Neurodegenerative Diseases, University of Toronto, Toronto, ON Canada; 2 Institute of Medical Science, University of Toronto, Toronto, ON Canada; 3 Division of Neurology, Toronto Western Hospital, University Health Network, 399 Bathurst St. WW5-449, Toronto, ON M5T 2S8 Canada; 4 Krembil Research Institute, University Health Network, Toronto, ON Canada; 5 Internal Medicine Board, Kuwait Institution for Medical Specialty (KIMS), Andalous, Kuwait; 6 Geriatric Medicine, McMaster University, Hamilton, ON Canada; 7 Interdepartmental Division of Critical Care Medicine, University of Toronto, Toronto, ON Canada; 8 Institute of Health Policy, Management and Evaluation, University of Toronto, Toronto, ON Canada; 9 Toronto General Hospital Research Institute, University Health Network, University of Toronto, Toronto, ON Canada

**Keywords:** Infectious diseases, Neurology

## Abstract

Long COVID is associated with neurological and neuropsychiatric manifestations. We conducted an observational study on 97 patients with prior SARS-CoV-2 infection and persisting cognitive complaints that presented to the University Health Network Memory Clinic between October 2020 and December 2021. We assessed the main effects of sex, age, and their interaction on COVID-19 symptoms and outcomes. We also examined the relative contribution of demographics and acute COVID-19 presentation (assessed retrospectively) on persistent neurological symptoms and cognition. Among our cohort, males had higher hospitalization rates than females during the acute COVID-19 illness (18/35 (51%) vs. 15/62 (24%); *P* = .009). Abnormal scores on cognitive assessments post-COVID were associated with older age (AOR = 0.84; 95% CI 0.74–0.93) and brain fog during initial illness (AOR = 8.80; 95% CI 1.76–65.13). Female sex (ARR = 1.42; 95% CI 1.09–1.87) and acute shortness of breath (ARR = 1.41; 95% CI 1.09–1.84) were associated with a higher risk of experiencing more persistent short-term memory symptoms. Female sex was the only predictor associated with persistent executive dysfunction (ARR = 1.39; 95% CI 1.12–1.76) and neurological symptoms (ARR = 1.66; 95% CI 1.19–2.36). Sex differences were evident in presentations and cognitive outcomes in patients with long COVID.

## Introduction

The global pandemic of coronavirus-19 (COVID-19) illness has been an unparalleled public health emergency worldwide. As of December 16th, 2022 the World Health Organization reported over 647 million confirmed cases of COVID-19 including 6.64 million deaths^1^. The presenting illness can range from asymptomatic to severe and fatal disease. The virus behind COVID-19 is the novel severe acute respiratory syndrome coronavirus 2 (SARS-CoV-2) which has been shown to gain entry into cells through the ACE2 receptor^2^. Even though COVID-19 most commonly presents with respiratory symptoms, ACE2 is found in other organ systems including cardiovascular, digestive, and nervous system, which can be the cause behind COVID-19 multi-organ damage^3^.

The most common initial symptoms of COVID-19 illness are fever, cough, fatigue, and loss of taste or smell; with other symptoms including sore throat, headache, aches and pains, diarrhea, rash, and red or irritated eyes^1^. The majority of SARS-CoV-2 infections result in mild disease, with reported incidence of severe disease and mortality to be 12.6–23.5% and 2.0–4.4%, respectively^4^. Predictors of severe COVID-19 illness were found to be male sex, increasing age and comorbidities including history of smoking, obesity, diabetes, hypertension, and cardiovascular diseases^5–7^.

Studies show that while some patients recover and resume normal functioning following a SARS-CoV-2 infection, others see symptoms remain for over 4 weeks—a condition now known as long COVID^8^. Reported incidence of long COVID varies among studies from 4.7 to > 90%^9–11^ and was more likely with increasing age, body mass index and female sex^11^. It has been reported that neuropsychiatric complaints of depression, stress, anxiety, and insomnia are common among long haulers^12–14^, with some studies reporting higher rates of post-traumatic stress disorder symptoms in patients with long COVID^15^. There is inconsistency in the literature with some studies finding neurological complaints in long COVID including fatigue, “brain fog” and memory complaints as the most prominent neurological symptoms^13^, while one study did not find neurological deficits in long haulers^12^. A large meta-analysis study reported that 12 or more weeks following the COVID-19 diagnosis, the proportions of individuals experiencing fatigue and cognitive impairment was 0.32 and 0.22, respectively^16^. Out of those studies that compared neurological and cognitive deficits among hospitalized and non-hospitalized COVID patients, hospitalizations and ICU admissions were associated with a higher frequency of memory complaints, fatigue, anxiety, depression, and sleep disturbances^13^.

Understanding the evolution of neurological and neuropsychiatric symptoms of long COVID is crucial for accurate patient prognosis. This study, therefore, aimed to characterize symptom history, neuropsychological, and neuropsychiatric functioning in a sample of patients with long COVID presenting with cognitive complaints. Moreover, we examined factors such as demographics, comorbidities, and initial symptom presentation for their ability to predict the prolonged symptom burden.

## Methods

### Participants

Ninety-seven consecutive patients who presented to the University Health Network (Toronto, Canada) memory clinic with cognitive complaints between October 15th, 2020 to December 9th, 2021 were included in this observational study. All patients had a previously confirmed diagnosis of COVID-19 through a positive result on a reverse transcriptase polymerase chain reaction (RT-PCR) assay of a specimen collected on a nasopharyngeal swab. An extensive clinical evaluation, including acute and persistent symptom burden and past medical history, was completed using a self-report questionnaire, and cognitive assessments were completed for all patients. The questionnaire to collect the symptom burden was used as previously described, with an additional review of system checklist added^17^. Cognitive functioning was assessed by the Toronto Cognitive Assessment (TorCA)^18^ or the Montreal Cognitive Assessment (MoCA)^19^. In brief, TorCA consists of 27 subtests within seven cognitive domains—orientation, immediate recall, delayed recall, delayed recognition, visuospatial function, working memory/attention/executive control, and language, as previously described^18^. Abnormal score on TorCA was determined by established cut-offs^18^, while abnormal score on MOCA was < 26^19^. A subset (N = 35) of participants had completed an MRI scan to check for brain abnormalities. Patients were diagnosed with mild cognitive impairment (MCI) if they scored below cut-offs on the MOCA or TorCA, patients were diagnosed with subjective cognitive impairment (SCI) if they had new onset cognitive complaints but scored within normal range on cognitive assessments. This study was approved by the Research Ethics Board of the University Health Network and written informed consent was obtained from all participants. All methods were carried out in accordance with relevant guidelines and regulations.

### Magnetic resonance imaging

A clinical MRI with susceptibility-weighted imaging was obtained. Abnormal MRI findings were quantified as the presence of brain hemorrhages and/or microhemorrhages.

### Statistical analyses

All descriptive data is presented as mean (standard deviation) for normally distributed variables and median (range) for non-normally distributed variables. Statistical analysis was completed using IBM SPSS Statistics v.28 (IBM Corp., Armonk, NY, USA) and R studio with R v4.1.3 (https://www.r-project.org/). Study participants were stratified into young adults (< 60 years old (y.o.) and older adults (60 + y.o.) groups. First, multiple linear regression was completed to assess for the main effects of sex, age group, and their interaction on past medical history and COVID-19 illness outcomes across the whole cohort. Next, differences in acute and persistent COVID-19 symptom domains were compared across age and sex groups, adjusting for combined past medical history, ICU admission status, and abnormal MRI findings. To control for possible confounders in cognitive outcomes, patients who were admitted to ICU during the acute COVID-19 illness and/or had abnormal MRI findings were excluded from further analysis on the effects of sex/age on cognitive domains and predictive models. Best subset regression with cross-validation procedure and the number of repetitions *t* = 1000 (“bestglm” package in R) was completed to analyze the predictive ability of variables of interest on persistent symptom burden and cognitive outcomes. Variables selected for predictive models included age, sex, duration of symptoms, relevant past medical history (i.e., history of psychiatric illness for prediction of persistent psychiatric symptoms and history of neurological conditions for prediction of persistent cognitive symptoms), and symptoms presented at initial COVID infection. Demographic variables such as age, sex, and relevant past medical history were always included in the final model and adjusted risk ratios (ARRs) and odds ratios (AORs) are reported.

## Results

### Cohort descriptors

**Figure 1:**
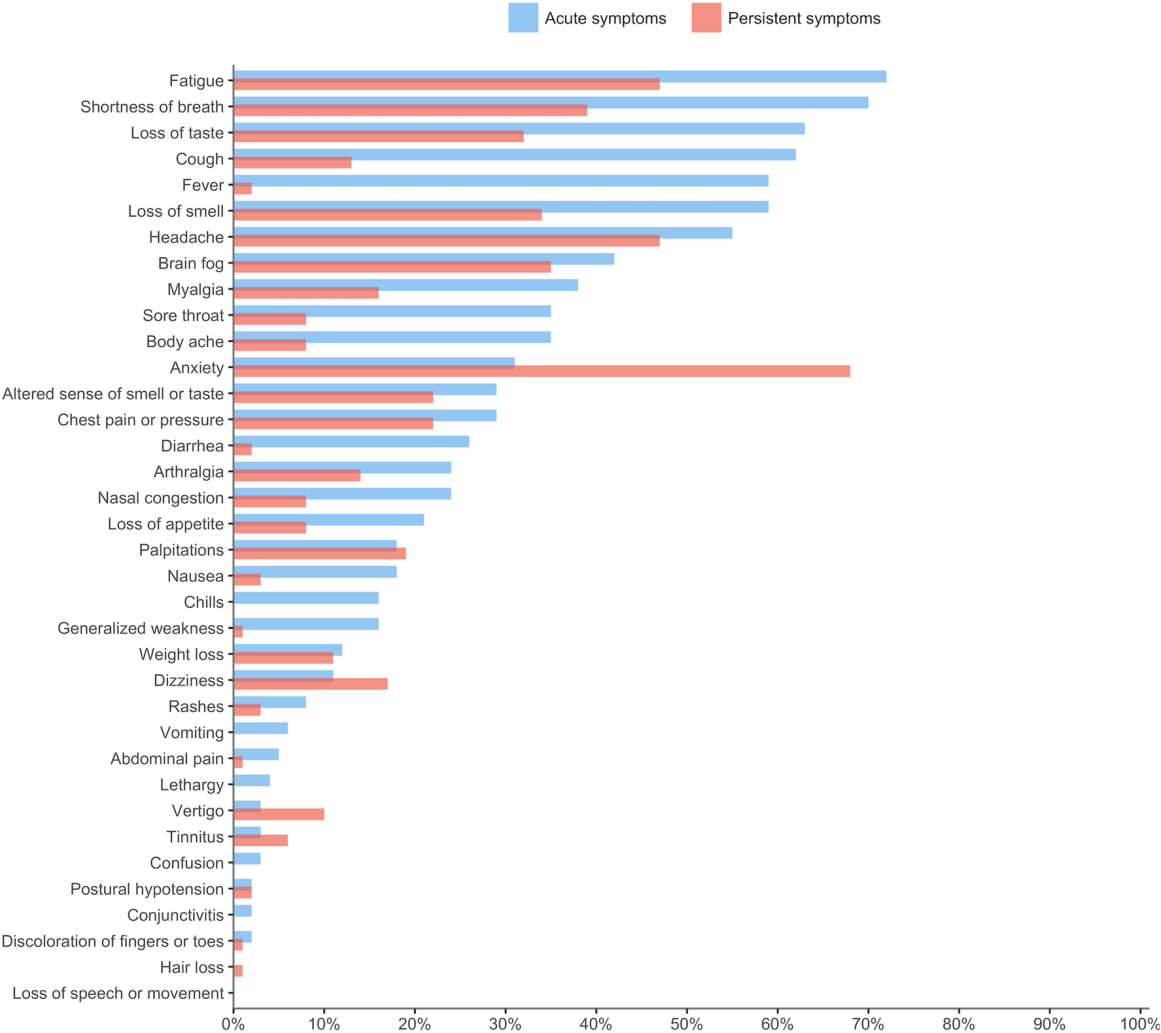
Percentage of self-reported acute and persistent symptoms for 97 long COVID patients. Symptoms are ordered based on the frequency of acute symptoms.

Ninety-seven patients (mean [range] age, 52 [27–83] years; 62 (64%) female) were included in this cross-sectional study. The mean symptom duration since the onset of acute symptoms to the assessment was 9.7 months (range: 3–21 months). Out of 97 patients, 19 (20%) were initially admitted to ICU because of acute COVID-19 illness and 16 (16% of total patients) out of ICU admitted patients were intubated. A subset of 35 (36%) patients completed MRI imaging and 7 (20%) out of those were found to have brain microhemorrhages. The percentage of self-reported acute and persistent symptoms is outlined in Fig. 1. The acute symptoms experienced by > 50% of patients in this cohort were fatigue (72%), shortness of breath (70%), loss of taste (63%), cough (62%), fever (59%), loss of smell (59%), and headache (55%). The majority of symptoms decreased in incidence following the resolution of acute COVID-19 illness except for anxiety, which increased from 31 to 68% post-acute COVID-19. Other less common symptoms that increased in incidence post-acute COVID-19 were dizziness (11% to 17%), vertigo (3% to 10%), tinnitus (3% to 6%), and hair loss (0% to 1%). The majority of patients [55 (59%)] returned to work post-COVID-19 illness. With respect to post-COVID-19 cognitive impairment diagnoses in this cohort, [75 (77%)] were diagnosed with SCI, and [20 (21%)] with MCI. Abnormal TorCA or MOCA scores were found in 23 (24.5%) patients in this cohort.

### Past medical history and COVID-19 outcomes

Past medical history, acute and persistent COVID-19 outcomes are outlined in Table 1. The most common past medical history conditions were psychiatric disorders (37%), heart disease (24%), and metabolic disorders (21%). Older adults had a higher incidence of heart disease compared to younger adults (12/26 (46%) vs. 11/71 (15%); *P* = 0.003). Females had a higher incidence of past psychiatric illness compared to males (28/62 (45%) vs. 8/35 (23%); *P* = 0.05). Males had a higher incidence of past metabolic disorders compared to females (12/35 (34%) vs. 8/62 (13%); *P* = 0.01), with the older male group having the highest incidence of metabolic disorders (8/12 (67%); *P* = 0.03). During initial COVID-19 illness, males had higher hospitalization rates (18/35 (51%) vs. 15/62 (24%); *P* = 0.009). Finally, older adults 60 + y.o. had the highest incidence of MCI diagnoses post-COVID-19 illness compared to younger adults < 60 y.o. (9/26 (35%) vs. 11/71 (15%); *P* = 0.05).

**Table 1:** Past medical history and COVID-19 acute illness outcomes stratified by age and sex.

	Total (N = 97)	Younger adults (< 60 y.o.)		Older adults (60 + y.o.)		*P* value		
		Female (N = 48)	Male (N = 23)	Female (N = 14)	Male (N = 12)	Age	Sex	Age x Sex
Past medical history, no. (%)								
Lung disease	15 (15%)	6 (13%)	3 (13%)	4 (29%)	2 (17%)	.3	.6	.5
Heart disease	23 (24%)	8 (17%)	3 (13%)	4 (29%)	8 (67%)	**.003**	.2	.09
Hematological disorders	4 (4%)	1 (2%)	1 (4%)	1 (7%)	1 (8%)	.3	.7	.8
Endocrine disorders	18 (19%)	6 (13%)	6 (26%)	2 (14%)	4 (33%)	.7	.09	.9
Rectal/prostate cancer	2 (2%)	0	0	0	2 (17%)	NA	NA	NA
Organ/bone marrow transplant	1 (1%)	1 (2%)	0	0	0	NA	NA	NA
Infectious disease	2 (2%)	0	2 (9%)	0	0	NA	NA	NA
Long term use of immunosuppressants/steroids	2 (2%)	2 (4%)	0	0	0	NA	NA	NA
Kidney disease	3 (3%)	2 (4%)	0	0	1 (8%)	NA	NA	NA
Liver disease	2 (2%)	1 (2%)	1 (4%)	0	0	NA	NA	NA
Psychiatric disorders	36 (37%)	22 (46%)	6 (26%)	6 (43%)	2 (17%)	.5	**.05**	.7
Neurological disorders	16 (16%)	9 (19%)	2 (9%)	3 (21%)	2 (17%)	.5	.4	.7
GI disorders	6 (6%)	2 (4%)	0	3 (21%)	1 (8%)	.9	.9	.9
Metabolic disorders	20 (21%)	7 (15%)	4 (17%)	1 (7%)	8 (67%)	.3	**.01**	**.03**
Bone disorders	6 (6%)	2 (4%)	1 (4%)	2 (14%)	1 (8%)	.3	.8	.7
Sleep disorders	16 (16%)	6 (13%)	3 (13%)	3 (21%)	4 (33%)	.1	.6	.6
Initial COVID-19 illness, no. (%)								
Hospitalized	33 (34%)	13 (27%)	12 (52%)	2 (14%)	6 (50%)	.4	**.009**	.5
Admitted to ICU	19 (20%)	7 (15%)	8 (35%)	1 (7%)	3 (25%)	.4	.06	.8
Intubated	16 (16%)	5 (10%)	8 (35%)	1 (7%)	2 (17%)	.3	.09	.7
Post COVID-19 outcome, No. (%)								
Returned to work	55 (59%)^a^	31 (65%)	14 (61%)	7 (50%)	3 (33%)^a^	.4	.09	.6
Post-COVID diagnosis								
Normal	2 (2%)	2 (4%)	0	0	0	NA	NA	NA
SCI	75 (77%)	38 (79%)	20 (87%)	10 (71%)	7 (58%)	**.05**	.8	.4
MCI	20 (21%)	8 (17%)	3 (13%)	4 (29%)	5 (42%)			
Abnormal TorCA/MOCA	23 (24%)^b^	10 (21%)^c^	4 (17%)	3 (23%)^c^	6 (55%)^c^	.1	.3	.1

Data is presented as No. (%). Binary logistic regression was used; COVID-19 outcome analysis was adjusted for combined past medical history. “NA” designation was used where low per-group patient number did not allow for statistical comparisons.

*NA* not applicable; *SCI* subjective cognitive impairment; *MCI* mild cognitive impairment; *TorCA* toronto cognitive assessment; *MOCA* montreal cognitive assessment.

^a^Three patients were retired and were not included in this calculation.

^b^Calculated out of total N = 94 patients that completed TorCA or MOCA assessment.

^c^One patient did not complete TorCA or MOCA. Significant values are in [bold].

### Reported acute and persistent symptoms after COVID-19

The burden of acute and persistent self-reported symptoms is outlined in Table 2. Older (60 + y.o.) males had the shortest symptom duration (*P* = 0.05). Females were at a higher risk of developing more acute COVID-19 symptoms (10 vs. 6 symptoms; *P* = 0.001) and more persistent COVID-19 symptoms (19 vs. 14 symptoms, *P* < 0.001), compared to males.

**Table 2:** Burden of self-reported acute and persistent symptoms stratified by age and sex.

	Total (N = 97)	Younger adults (< 60 y.o.)		Older adults (60 + y.o.)		*P* value		
		Female (N = 48)	Male (N = 23)	Female (N = 14)	Male (N = 12)	Age	Sex	Age × Sex
Acute symptoms, median (Min–Max)								
Total symptoms	9 (0–19)	11 (0–16)	7 (0–17)	10 (0–19)	6 (4–9)	.7	**.001**	.3
Persistent symptoms, median (Min–Max)								
Time from acute symptom onset to clinic visit, months	9 (3–21)	9 (3–18)	9 (3–21)	9 (5–20)	8 (4–18)	.6	.06	**.05**
Physical symptoms	1 (0–6)	2 (0–6)	1 (0–4)	1 (0–4)	1 (0–3)	.07	.5	.8
Short term memory symptoms	3 (0–4)	3 (0–4)	3 (0–4)	3 (0–4)	2 (0–3)	.2	.1	1
Executive function symptoms	3 (0–6)	3 (0–5)	3 (0–5)	3.5 (2–6)	2.5 (0–4)	.8	.1	.4
Language symptoms	2 (0–5)	2 (0–5)	2 (0–4)	2 (0–5)	2 (0–4)	.7	.3	1
Visuospatial symptoms	0 (0–2)	0 (0–2)	0 (0)	0 (0–1)	0 (0)	NA	NA	NA
Personality symptoms	0 (0–3)	0 (0–3)	0 (0–2)	0 (0–3)	0 (0–1)	NA	NA	NA
Neuropsychiatric symptoms	5 (0–9)	5 (0–8)	4 (0–9)	4 (1–9)	4 (0–8)	.9	.3	.9
Neurological symptoms	3 (0–11)	3 (1–8)	2 (0–7)	3 (0–11)	2 (0–7)	.8	.1	.9
Total symptoms	18 (2–37)	20 (2–33)	15 (2–25)	16 (7–37)	13.5 (3–25)	.08	** < .001**	.4

Data presented as Median (Min–Max). Symptom burden is quantified as number of self-reported symptoms per person. Negative binomial regression with log link to control for overdispersion adjusted for ICU admission status, combined past medical history, and abnormal brain MRI findings was used. “NA” designation was used where low per-group patient number did not allow for statistical comparisons. NA, not applicable. Significant values are in [bold].

### Characteristics of the subset of patients with ICU admission

Overall, 19 (20%) out of 97 patients were admitted to the ICU during their acute COVID-19 illness. There was a trend for a higher risk of ICU admission among males in comparison to females (11/35 (31%) vs. 8/62 (13%); *P* = 0.06), see Table 1. The patients with prior ICU admission were similar to those with no ICU admission history in age (mean [S.D.]: 53^8^ vs. 52^17^ years; *P* = 0.6), time since acute symptom onset (median [range]: 9^4–15^ vs. 9^1,3–20^ months;* P* = 0.5), and combined number of past illnesses (median [range]: 2 [0–9] vs. 2 [0–8]; *P* = 0.3). There were no significant differences between patients with and without ICU admissions in total TorCA/MOCA scores (calculated as obtained/max. score ratio; *P* = 0.1), total TorCA scores alone (*P* = 0.12), or any of TorCA neuropsychological domain scores (all *P* > 0.2), adjusted for years of education, symptom duration, and combined past medical history. However, when looking into patients who were intubated [16/97 (16%)]—the subset that had intubation had significantly lower TorCA/MOCA scores (mean [S.D.]: 0.85 [0.07] vs. 0.90 [0.06]; *P* = 0.03), compared with patients with no intubation history, controlled for years of education, symptom duration, and combined past medical history. Patients with and without intubation history were similar in age (mean [S.D.]: 53^8^ vs. 52^11^ years; *P* = 0.9), time since acute symptom onset (median [range]: 9^5–15^ vs. 9^1,3–20^ months; *P* = 0.9), and combined number of past illnesses (median [range]: 2 [0–9] vs. 2 [0–8]; *P* = 0.5).

### Characteristics of the subset of patients with microhemorrhages

Out of 35 patients who completed MRI seven (20%) were found to have microhemorrhages. Among the seven patients with microhemorrhages were four women with no history of ICU admission or intubation, and three men who were all previously admitted to ICU and intubated. The four women with microhemorrhages had the following diagnoses: 3 SCI and 1 MCI. Of the three men with microhemorrhages, all three were diagnosed with SCI.

### Neuropsychological scores among patients with no ICU admission and normal brain MRI

To control for possible confounding effects of brain microhemorrhages and/or ICU admission on neuropsychological scores, only patients with normal brain MRI and no COVID-19 related ICU admission history were included in the neuropsychological scores analysis (see Table 3). Older than 60 years old males were at the highest risk for lower scores on TorCA or MOCA (*P* = 0.05). Higher age alone was a risk factor for lower delayed memory recognition scores (*P* = 0.05). No other effects of sex or age were found in measures of memory, attention, executive and visuospatial function, or language.

**Table 3:** Neuropsychological assessment scores stratified by age and sex.

	Total (N = 72)	Younger adults (< 60 y.o.)		Older adults (60 + y.o.)		*P* value		
		Female (N = 38)	Male (N = 15)	Female (N = 10)	Male (N = 9)	Age	Sex	Age × Sex
TorCA or MOCA, obtained/max. score	0.9 (0.1)	0.9 (0.1)	0.9 (0.1)	0.9 (0.1)	0.8 (0.1)	.2	.09	**.05**

	Total (N = 45)	Female (N = 21)	Male (N = 7)	Female (N = 8)	Male (N = 9)	Age	Sex	Age × Sex
TorCA domain scores, Mean (S.D.)								
Memory orientation	11.9 (0.4)	12.0 (0.2)	12.0 (0)	11.9 (0.4)	11.8 (0.7)	.2	.8	.5
Normal	44 (98%)	21 (100%)	7 (100%)	8 (100%)	8 (89%)	NA	NA	NA
Borderline/impaired	1 (2%)	0	0	0	1 (11%)			
Memory immediate recall	19.6 (4.4)	20.3 (5.4)	19.7 (1.8)	20.3 (3.5)	17.6 (3.8)	.4	.2	.4
Normal	23 (51%)	10 (48%)	2 (29%)	6 (75%)	5 (56%)	.1	.2	1
Borderline/impaired	22 (49%)	11 (52%)	5 (71%)	2 (25%)	4 (44%)			
Memory delayed recall	19.1 (4.1)	19.8 (4.2)	19.0 (5.9)	18.8 (2.9)	17.9 (3.3)	.4	.5	1
Normal	35 (78%)	16 (76%)	5 (71%)	6 (75%)	8 (89%)	.5	.7	.5
Borderline/impaired	10 (22%)	5 (24%)	2 (29%)	2 (25%)	1 (11%)			
Memory delayed recognition	20.0 (2.3)	20.1 (1.4)	21.4 (3.9)	19.1 (3.0)	19.7 (1.4)	**.05**	.2	.5
Normal	27 (60%)	12 (57%)	3 (43%)	6 (75%)	6 (67%)	.2	.5	.9
Borderline/impaired	18 (40%)	9 (43%)	4 (57%)	2 (25%)	3 (33%)			
Visuospatial function	30.2 (2.6)	30.0 (3.0)	30.9 (1.6)	30.5 (1.4)	29.8 (3.3)	.7	1	.4
Normal	41 (91%)	18 (86%)	7 (100%)	8 (100%)	8 (89%)	NA	NA	NA
Borderline/impaired	4 (9%)	3 (14%)	0	0	1 (11%)			
Working memory/attention/executive function	94.4 (16.4)	95.8 (13.2)	95.0 (14.5)	98.4 (16.0)	87.0 (24.3)	.6	.2	.3
Normal	9 (20%)	4 (19%)	2 (29%)	2 (25%)	1 (11%)	.1	.2	1
Borderline/impaired	36 (80%)	17 (81%)	5 (71%)	6 (75%)	8 (89%)			
Language	95.0 (14.1)	93.6 (11.8)	97.0 (9.1)	96.8 (12.3)	95.1 (23.1)	.8	1	.5
Normal	41 (91%)	18 (86%)	7 (100%)	7 (87.5%)	9 (100%)	NA	NA	NA
Borderline/impaired	4 (9%)	3 (14%)	0	1 (12.5%)	0			
TorCA total score	290.3 (22.3)	291.6 (24.3)	295.0 (22.6)	295.6 (21.3)	278.8 (16.3)	.5	.2	.1

Data presented as Mean (S.D.). Patients admitted to ICU and/or with abnormal brain MRI findings are excluded. Linear and binary regressions adjusted for combined past medical history and symptom duration. All neuropsychological scores where z-scores were not computed were additionally adjusted for years of education. Significant values are in [bold].

### Factors associated with COVID-19 outcomes in patients with no ICU admission and normal brain MRI

Abnormal TorCA or MOCA scores post-COVID were associated with older age (AOR = 0.84; 95% CI 0.74–0.93; *P* = 0.002) and acute brain fog symptoms during initial illness (AOR = 8.80; 95% CI 1.76–65.13; *P* = 0.02). Female sex (ARR = 1.42; 95% CI 1.09–1.87; *P* = 0.03) and acute shortness of breath (ARR = 1.41; 95% CI 1.09–1.84; *P* = 0.05) were associated with a higher risk of experiencing more persistent short term memory symptoms. Female sex was the only predictor associated with more persistent executive function symptoms (ARR = 1.39; 95% CI 1.12–1.76; *P* = 0.005) and more persistent neurological symptoms (ARR = 1.66; 95% CI 1.19–2.36; *P* = 0.001); while patients experiencing anxiety during acute COVID illness were at risk of developing a higher burden of persistent neuropsychiatric symptoms (ARR = 1.28; 95% CI 1.01–1.63; *P* = 0.006).

### Neuropsychiatric outcomes in patients with no ICU admission and normal brain MRI

The persistent self-reported neuropsychiatric symptoms were prominent in this cohort with a median of 5 neuropsychiatric symptoms per patient. The highest incidence was seen in anxiety [54/74 (73%)], followed by irritability [53/74 (72%)], trouble staying asleep [48/74 (65%)], emotional lability [42/74 (57%)], and low mood [39/74 (53%)]. There were no differences in neuropsychiatric symptom incidence between sex and age groups, adjusted for past history of psychiatric disorders.

## Discussion

In this study, we described the persistent neurocognitive and neuropsychiatric signs and symptoms among an observational cohort of 97 patients who were previously positive for a SARS-CoV-2 infection. The most common acute symptoms experienced in this cohort were fatigue, shortness of breath, loss of taste, cough, fever, loss of smell, and headache. The majority of symptoms decreased in frequency after the resolution of acute COVID-19, while others such as anxiety, headache, and fatigue persisted. Interestingly, the incidence of anxiety greatly increased in long COVID in comparison to acute COVID illness, which is consistent with other literature reporting high incidence of neuropsychiatric symptoms following COVID-19^12,13^. Males were at a higher risk of being hospitalized and admitted to ICU which shows a potential for a more severe acute illness, while females were at a higher risk for a higher burden of self-reported acute and persistent symptoms. Furthermore, our cohort of long COVID patients with cognitive complaints was mostly female (64%).

Male sex was previously reported as being associated with more severe acute COVID-19 illness^20,21^, however, the reasons for this sex disparity are less clear. Hypotheses for why male sex is associated with higher morbidity and more severe COVID-19 illness range from effects of lifestyle (higher smoking rates in males leading to a higher rate of comorbidities) and a stronger immune response in females offering protection against severe COVID-19. Another hypothesis suggests that estrogen hormone and location of ACE2 gene on the X chromosome leads to overexpression of ACE2 in females^22^. As COVID-19 has been shown to downregulate ACE2 expression leading to angiotensin imbalance and multisystem disfunction^23–25^, the overexpression of ACE2 in females offers additional protection against severe illness^22^. A stronger immune response and physiological differences may, however, play a role in prolonging disease manifestations in females as an emerging body of research reports female sex as a risk factor for developing long COVID^26–28^. Finally, the overall rates for post-COVID-19 cognitive diagnoses in our study were largely SCI (77%), followed by MCI (21%), with older age being a risk factor for being diagnosed with MCI.

The subset of patients that were admitted into ICU during acute COVID-19 illness were similar in demographics and neuropsychological scores to patients with no prior ICU admission status. However, the subset of patients who were intubated had lower cognitive scores compared to patients with no intubation history. Our results are consistent with past studies showing that ICU admission and hospitalization status are associated with worse cognitive outcomes in patients with COVID-19 and other critical illnesses^29,30^. Although critical illness and severe COVID-19 illness were reported to be associated with brain microhemorrhages^31–34^, we were surprised to find a similar number of microhemorrhages in mild COVID cases. In our cohort the rates of SCI and MCI were similar between patients with and without microhemorrhages, making the effects of microhemorrhages on cognitive outcomes unclear. It is important to mention that the timing and the causality of the microhemorrhages found in our patient cohort are unknown and the absence of a COVID-negative comparison group limits further interpretation of these findings.

In the patients with no prior ICU admission history and normal brain MRI scan the global cognition was lowest in older (60 y.o.) males. Older males in this cohort had the shortest duration since the acute symptom onset which could suggest that this group needed more time for their cognition to improve or the worse functioning could be a direct consequence of a more severe COVID-19 presentation in males, despite this analysis being done with the most severe COVID-19 cases excluded. An alternative explanation for higher rates of cognitive decline among older patients and older males could be that the SARS-CoV-2 infection unmasked a pre-existing cognitive impairment. When we examined risk factors associated with impaired global cognitive function (rather than a continuous score)—the risk for impaired cognition was higher in older patients and those who experienced brain fog as the initial symptom. Interestingly, the presence of acute anxiety posed a higher risk for more persistent neuropsychiatric symptoms, regardless of the past history of neuropsychiatric illness. This could be explained by an already heightened incidence of neuropsychiatric symptoms as a result of the pandemic, compounded with the fear of the illness itself^35^. The need for health information and the perceived impact of the pandemic were found to mediate the relationship between the symptoms resembling a SARS-CoV-2 infection and the adverse mental health outcomes like depression and anxiety. Specifically, conflicting health information like differences in proposed mask policies and unconfirmed rumors about the COVID-19 pandemic might heighten the perceived impact of the pandemic and increase its negative impacts on mental health^36^. Another explanation could be that as the virus attacks the limbic system, it leads to heightened acute and persistent neuropsychiatric symptoms^37^.

In our cohort, we examined risk factors associated with specific symptom burden and found that females had a higher risk for more persistent deficits in executive function and neurological symptoms (i.e., headache, vertigo, vision and hearing changes, weakness, tremors, among others); while female sex together with shortness of breath during acute illness were found to be a risk factor for more persistent short-term memory symptoms. This provides additional evidence that females are more vulnerable to prolonged effects following SARS-CoV-2 infection. At the same time, this raises the possibility that mild hypoxia may be contributing to these ongoing memory complaints. Evidence from other cohorts like elite breath-hold divers and patients with sleep apnea shows that mild instances of hypoxia lead to persistent short-term memory impairments^38,39^. A recent study examining long-term cognitive impairments following COVID-19 reported that patients with hypoxemic pneumonia showed worse memory than outpatients^40^. Even though we excluded patients with severe acute COVID-19 from our analysis, we still found a relationship between shortness of breath during acute COVID and persistent memory issues. This suggests that even mild COVID-19 illness not requiring hospitalization may cause lung damage sufficient to induce mild hypoxia leading to persistent memory complaints.

In the older population one needs to consider the possibility of neurodegeneration being accelerated or the cognitive symptoms amplified. There is evidence for increased levels of neurodegenerative markers in serum and cerebrospinal fluid in COVID-19 patients^41,42^, and worsening parkinsonian symptoms following COVID-19 illness in patients with Parkinson’s disease^43,44^; finally, patients aged 51–81 y.o. showed a greater cognitive decline than controls following a SARS-CoV-2 infection^45^.

The global pandemic had significant effects on the mental health of the general population, raising the incidence of neuropsychiatric complaints globally^35^. The incidence of persistent anxiety in our cohort was found to be 73%, which is higher compared to the reported anxiety rates in the general population during the pandemic (22–41%). The rate of low mood in our cohort was 53% which was on the higher end of the depression rates reported in the general population during the pandemic (20–52%)^46–49^. The incidence of patients who had trouble staying asleep was 65%, which was higher than the reported incidence of insomnia of 18–23% in the general population during the pandemic^49^. This suggests that experiencing persistent COVID symptoms adds additional emotional distress on top of that experienced as a result of the pandemic. Despite a higher prevalence of history of psychiatric illness in females, we found no significant differences in neuropsychiatric scores between sexes or age groups suggesting that the general mental health issues related to the pandemic maybe be masking any COVID-illness-related changes.

This study has several limitations including possible referral bias in our patient sample, small sample size, and high heterogeneity of our cohort with regards to age, symptom duration, and severity of COVID-19 illness. The patient cohort for our study was recruited during the first phase of the COVID-19 pandemic in the pre-vaccine era, and therefore our findings should be interpreted with caution. Only a subset of our cohort had completed brain MRI scans therefore we could not fully control for the presence of underlying brain abnormalities on cognitive outcomes. The symptom and history of presenting illness were all based on self-reports, which limits objectivity. The lack of a COVID-negative group comparison limits the interpretation of our findings. Future studies would need to be conducted in larger cohorts to validate our results and prediction models.

In conclusion, males were found to have a more severe acute illness while more females developed long COVID neurological issues. Increased incidence of neuropsychiatric complaints in patients with long COVID continues to be an area of concern where further research is needed. The study also highlights that sex and age need to be factored into any analyses as COVID-19 has brought to the forefront the need for personalized medicine for both diagnosis and prognostication.

## Data Availability

Data in a deidentified format will be made available by request to the corresponding author.
